# Occurrence and antimicrobial susceptibility patterns of *Escherichia coli* and *Escherichia coli* O157 isolated from cow milk and milk products, Ethiopia

**DOI:** 10.1038/s41598-023-43043-8

**Published:** 2023-09-25

**Authors:** Edilu Jorga Sarba, Wakuma Wirtu, Endrias Zewdu Gebremedhin, Bizunesh Mideksa Borena, Lencho Megersa Marami

**Affiliations:** 1 Department of Veterinary Sciences, School of Veterinary Medicine, Ambo University, P.O. Box 19, Ambo, Ethiopia; 2 Department of Biology, Addis Ababa Education Office, Addis Ababa, Ethiopia; 3 Department of Veterinary Laboratory Technology, School of Veterinary Medicine, Ambo University, P.O. Box 19, Ambo, Ethiopia

**Keywords:** Biological techniques, Microbiology, Diseases, Medical research

## Abstract

*Escherichia coli* is a major foodborne pathogen worldwide. This study was conducted to assess the prevalence, risk factors, and antimicrobial susceptibility of *E. coli* and *E. coli* O157 in milk and milk products and hygienic practices in West Shoa, Oromia, Ethiopia. Five hundred fifty-six milk samples comprising 421 udder milk, 57 bulk tank milk, and 78 milk products were investigated. Moreover, a questionnaire was administered to 145 participants to assess hygienic practices. A standard microbiological procedure was used to detect *E. coli* and *E. coli* O157. The Kirby Bauer disc diffusion method was used to test the antimicrobial susceptibility of the isolates. Petri film plates were used to enumerate the coliform in raw bulk tank milk. Farm and animal-level *E coli* prevalence were estimated and association with risk factors was assessed. *Escherichia coli* was detected in 33.8% (95% confidence interval (CI) 29.9–37.9%) of the samples, of which only one isolate (0.2%) was *E. coli* O157. *Escherichia coli* contamination was higher in bulk tank samples (47.4%; 95% CI 34.0–61.0%) than in udder milk (34.7%; CI 30.1–39.4%), cottage cheese (27.0%; 95% CI 14.6–43.9%), and yoghurt 10.5% (95% CI 2.9–24.8%). For the animal-level *E. coli* contamination, only the study area was identified as a risk factor. Risk factors such as types of milk containers, udder washing practices, hygiene, and management systems were associated with both farm-level and milk products *E. coli* contamination. Most (59.0%) of the farmers practice udder washing only before milking and the remaining did not practice udder washing at all. The mean coliform count in raw bulk tank milk was higher than the international standard (4.09 log10 CFU/ml). All the 42 *E. coli* isolates tested were multidrug resistant. The occurrence of *E. coli* is high, while that of *E. coli* O157 is too low. The milk-handling practices are poor in the study areas. The high prevalence of *E. coli*, as well as the high coliform count and higher multi-drug resistance may pose risk to public health and food safety. Therefore, proper hygienic practices throughout the milk chain as well as rational drug use are advised.

## Introduction

Foodborne diseases are a major public health concern globally with developing nations bearing the majority of the burden^1^. *Escherichia coli* (*E. coli*) is one of these bacteria that is commonly linked to foodborne illness. *E. coli* is a Gram-negative, non-spore-forming, flagellated, rod-shaped, and facultative anaerobic bacterium belonging to the family *Enterobacteriaceae*. Most *E. coli* strains are harmless, however, the *E. coli* O157 strain is a significant foodborne pathogen with a low infective threshold and high resistance to treatment^2^.

Cattle are the primary source of *E. coli* O157, but sheep and possibly goats may also be involved^3^. Human diseases caused by *E. coli* O157 have been linked to ground beef, raw milk, dairy products, vegetables, unpasteurized fruit juices, water, and direct contact with ruminant feces^4^. Milk or dairy products are nutritious foods that billions of people consume every day^5^. Ingestion of raw milk and milk products is one of the sources for foodborne illnesses^6,7^. Hence, the detection of *E. coli*/*E. coli* O157 from milk and milk products is an indicator of a possible public health risk. The source of *E. coli* O157:H7 in milk and milk products could be the mammary gland, unhygienic milk handling, milk processing, and marketing^8^.

Ethiopia, as developing country encounters problems of unhygienic production and consumption of foods of animal origin such as milk. Unhygienic milk handling could result in contamination of milk with feces, manure, and poor-quality water in dairy farms^9^. The potential factors for the contamination of milk and dairy products with *E. coli* and *E. coli* O157 could therefore be the poor hygienic milking practice, farm environment, milkers’ hand, and milk handlers and containers. Recent studies reported the occurrence of *E. coli* and *E. coli* O157:H7 in milk and milk products from dairy farms, milk vendors and supermarkets in different parts of Ethiopia^10–12^. *Escherichia coli* and *E. coli* O157 was also isolated from dairy farm environmental samples such as feces and water in Ethiopia^11,13^. The isolation of this pathogen from milk and dairy farm environment is a potential factor and source of infection for humans consuming raw milk and milk products.

Diarrheal disease caused by contaminated foods accounts for 33% of the population in developed countries and about 70% of the population in developing countries. Diarrhea is one of the causes of childhood illness and death in developing countries. Pathogenic intestinal *E. coli* caused 8–10% of cases of diarrhea in children and *E. coli* O157:H7 causes 2,801,000 serious illnesses each year, with a global incidence rate of 43.1 cases per 100,000 person-years that includes all of Africa. The annual mortality rate in Africa, Asia, and America is around 4.6–6 million people^14^.

Antimicrobial resistance has been linked to the excessive use of antimicrobial drugs in food processing, in animals and humans; however, little attention has been given^15,16^. Studies show that multi-drug resistance in *E. coli* is a major concern worldwide; nevertheless, there were limited studies on this aspect particularly from developing countries^17,18^.

In Ethiopia, as in other countries, foodborne diseases are frequently unreported, and there is no surveillance program for foodborne pathogens. Likewise, it is difficult to demonstrate the extent of contamination of milk and milk products, which is a challenge to food safety^19^. Foodborne infections continue to be a major public health concern in Ethiopia because of the poor sanitary conditions, malnutrition, and lack of adequate medical services 16. Several reports from Ethiopia and other countries showed higher multidrug resistant *E. coli* and *E. coli* O157 H7 in milk samples^12,13,20^.

The available research findings revealed that pathogenic *E. coli* such as *E. coli* O157:H7 is a potential food-borne pathogen in humans and multidrug resistance is a major problem. Hence, studies targeting the occurrence, antimicrobial resistance, hygienic practices and potential risk factors for contamination of milk and milk products with *E. coli* and *E. coli* O157 are important as there is limited information particularly in the study area, despite the habit of consumption of raw milk and milk products. This research was therefore intended to investigate the prevalence, associated risk factors, and antimicrobial susceptibility patterns of *E. coli* and *E. coli* O157 isolates in milk and milk products; and to assess the hygienic quality of raw milk.

## Materials and methods

### Description of the study areas

This study was conducted in West Shoa Zone of Oromia region, Ethiopia. In this zone, crop-livestock mixed farming system is a common agricultural practice. The cattle population in the zone is estimated at 2,294,593, most of which are indigenous cattle breeds that are managed under extensive system, of which 472,138 and 123,682 are dairy cows either crossbred or exotic cattle breeds managed under intensive or semi-intensive system^21^. The towns such as Holota, Ambo and Bako are typical representatives of market-oriented smallholder dairy production using cross breed dairy cattle in urban and peri-urban areas Ethiopia.

The three selected towns: namely, Holeta, Ambo, and Bako, are found 29 km, 114 km, and 251 km West of Addis Ababa, the capital of Ethiopia, respectively. Holeta town is the administrative center of Welmara district. The town is located at a longitude and latitude of 9° 3′ N and 38° 30′ E, respectively, at an altitude of 2391 m.a.s.l, has a mean temperature of 15.9 °C and rainfall of 1134 mm. Holeta is one of the potential dairy production areas in central highland of Ethiopia serving as milk shade to Addis Ababa creating a better market access and fresh milk sale as a major source of income for dairy producers. The dairy farms in the town have relatively better access to improved management, and veterinary service. During the study period, there were about 668 crossbred lactating cows in the town producing approximately 7014 L of milk per day. The town has a total human population of 60,256 of which 29,526 are males and the rest 30,730 are females^22^.

Ambo is the administrative center of the West Shoa Zone of the Oromia region located at latitude and longitude of 8° 59′ N and 37° 51′ E, respectively. It is situated at an altitude of 2100 m above sea level (m.a.s.l.), mean temperature of 18.6 °C and mean rainfall of 1012 mm and has midland the weather condition. There are relatively fewer large scale dairy farms and substantial number of small holder dairy farms that provide fresh milk to the community of the town. The access to improved dairy management and veterinary service is relatively lower than Holeta. During the study period, there were around 580 total lactating crossbred cows that produce approximately 6090 L of milk per day. The town has a total human population of 117,649 of which 57,650 are males and the rest 59,999 are females^22^.

Bako town is the administrative center of Bako Tibe district in the West Shoa zone located at the longitude and latitude of 9° 08′ N and 37° 09′ 0″ E, respectively. Bako is situated at an altitude of 1650 m.a.s.l. with a mean temperature of 21.2 °C and rainfall is 886.5 mm. Bako is located at the western border of the zone with relatively warm weather conditions. There are fewer small dairy farms that provide fresh milk to the community of the town and the access to improve dairy management and veterinary service is relatively lower. There are about 50 lactating cows in the town producing approximately 525 L of milk per day. The town has an estimated population of 18,641 of whom 9370 are males and 9271 are females^22^.

### Study design

A cross-sectional study was conducted from November 2018 to June 2019 to investigate the prevalence, risk factors, and antimicrobial resistance of *E. coli* and *E. coli* O157 in milk and milk products and assess the hygienic status of cow milk.

### Sample size determination

The sample size was calculated using Thrusfield^23^ single population proportion formula, considering the 33.9% expected prevalence from Assossa, northwest Ethiopia^24^, 95% confidence interval, and 5% absolute precision. As a result, the calculated sample size for the animal-level milk sample (344) was increased to 421. In addition, 57 milk samples from the bulk tank at the farm level and 78 milk product samples (40 cottage cheese and 38 yoghurt prepared traditionally) were included. Accordingly, the total samples size comprising cow level milk sample, bulk tank milk sample and milk by product sample was 556.

### Sampling techniques and procedures

The three district towns were selected purposely from West Shoa Zone of Oromia region, Ethiopia depending on the availability of dairy cattle farms, agro-ecology, and accessibility. Due to the absence of recorded data on dairy farmers in the study area, first a baseline survey was conducted to identify the total number of dairy farms, farm size, farming system, number of lactating cows, and the hygienic status in the study areas. Dairy farms were categorized based on their herd size into small-scale- < 10 animals, medium-scale-10 to 50 animals, and large-scale commercial farms- > 50 animals) as per Megersa et al.^25^. Stage of lactation was classified as early, middle, and late, and the number of lactating cows was grouped as small, medium, and large. The hygienic status of the farm environment, and milk and milk products containers were rated as good when gross dirt and unpleasant smell were absent, poor when gross dirt and smell are present or fair when it is in-between.

Considering the number of the list of lactating cows in the three study towns as a sampling frame, the calculated sample size (421 lactating cows) was distributed proportionally to the three towns as (221 for Holeta, 150 for Ambo, and 50 for Bako) and the milk product samples (78) were distributed accordingly (36 Holeta, 30 Ambo and 12 from Bako). These 421 milk samples were collected from 67 farms and the number of cows sampled per farm ranged from 3 to 15 depending on the number of milking cows in the farms. A simple random sampling technique was applied to select the lactating cows in each town except for Bako where all the available lactating cows were sampled.

From the selected lactating cows, about 50 mL of raw udder milk was collected aseptically from a cow’s teats (composite milk from all functional teats). About 50 mL of milk samples from the bulk tank/collection center and about 50 g of cottage cheese from the market and 50 g of yoghurt from the cafeteria were collected. The samples were transported to the Ambo University Zoonosis and Food Safety Research Laboratory in an icebox with ice packs for bacteriological analysis. The examination of samples was conducted upon arrival at the laboratory.

### Questionnaire survey

A pre-tested questionnaire survey and observation checklists were used as a tool to gather information on the hygienic practices at the dairy farms, milk collection centers, cafeterias, and milk product sellers in the open markets. Accordingly, information regarding hygienic practices of milking, milk handling, storage, and transportation, duration of transportation, knowledge regarding contamination of milk with *E. coli*, and the associated risk factors were collected.

### Microbial analysis

#### Isolation and identification of *E. coli* and *E. coli* O157

About 25 mL of raw milk, 25 g of cottage cheese, and yoghurt samples (separately) were diluted in buffered peptone saline water (BPW) (225 mL). Each BPW enriched sample was incubated at 37 °C for 24 h. All pre-enriched samples were inoculated on MacConkey agar (SRL. Pvt. Ltd., Mumbai, India) at 37 °C for 24 h. Typical pink colonies on MacConkey agar were stained using Gram’s stain to observe their staining and morphological characteristics. Then, suspected colonies of *E. coli* were transferred to Eosin-Methylene-Blue (EMB) agar (SRL. Pvt. Ltd., Mumbai, India). The colonies with metallic sheen on EMB agar are typical *E. coli*. Additional confirmatory biochemical tests (indole production, methyl-red, Voges-Proskauer, and citrate utilization) and fermentation of lactose and glucose using triple sugar iron agar were considered. The putative *E. coli* colony was transferred to sorbitol MacConkey agar (Oxoid Ltd., Hampshire, England) to differentiate the inability to ferment sorbitol, which is the characteristic of *E. coli*. Then, non-sorbitol fermenting colonies were transferred to nutrient agar for serological testing^26^.

### Serological test

Sorbitol-negative colonies were serologically confirmed by using an *E. coli* O157 latex agglutination assay containing latex particles coated with antibodies specific to *E. coli* O157 (Abraxis LLC, USA). The Dry spot *E. coli* O157 latex test was confirmed by agglutination of *E. coli* strains possessing the O157 serogroup antigen. One drop of saline was dispensed to the small ring (at the bottom of each oval) in both the test and control reaction areas ensuring that the liquid did not mix with the dried latex reagents. Using a sterile single-use plastic loop, a portion of the colony to be tested was selected and carefully mixed in the saline drop until the suspension was smooth. Then, using a paddle the suspension was mixed into the dry latex spots until completely suspended and spread to cover the reaction area. The test card was picked up, stirred for up to 60 s, and looked for agglutination under normal lighting conditions. A result was considered positive if agglutination of the latex particles occurs within 1 min. This indicates the presence of *E. coli* O157. A negative result was recorded if no agglutination occurs, and a smooth blue suspension remains after a minute.

### Enumeration of the coliform

Twenty-five mL of raw milk from bulk was diluted in 225 mL buffered peptone saline water (SRL Pvt. Ltd., Mumbai, India) and serial dilution of 10^−1^, 10^−2^, 10^−3^, and 10^−4^ was applied to quantify the coliform bacteria to assess the hygienic quality of the raw milk. The diluted milk samples (1 mL/dilution) were dispensed on the 3 M™ Petri films (3 M, USA) plates and incubated for 24–48 h at 37 °C. After 24 h of incubation, the colonies grown on the films were counted by dividing the films into four quarters to facilitate the counting. The colonies in one quarter were counted and multiplied by four and by dilution to get the total colonies per dilution. Colonies greater than 100 in one quarter were rejected as too many to count and the average load of countable dilutions was taken and converted to log10^27^.

### Antimicrobial susceptibility testing

The Kirby–Bauer disc diffusion method was used to determine the antimicrobial susceptibility profile of sorbitol-negative *E. coli* strains^28^. Accordingly, Mueller–Hinton agar plates (HiMedia, India) were prepare and commonly used antimicrobials were considered. These antimicrobials include azithromycin (AZM) (30 μg), ceftriaxone (CTR) (5 μg), gentamycin (GEN) (10 μg), norfloxacin (NOR) (10 μg), tetracycline (TET) (30 μg), ciprofloxacin (CPR) (5 μg), co-trimoxazole (COT) (30 μg), nitrofurantoin (NIT) (300 μg), ceftazidime (CAZ) (30), nalidixic acid (NAL) (30 μg), amikacin (AMK) (30 μg), amoxicillin (AMX) (2 μg), and ampicillin (AMP) (10 μg). Then, isolates with bacterial suspension equivalent to 0.5 McFarland standards, were uniformly streaked on Mueller Hinton agar, and plates with antimicrobial discs (Oxoid, UK) were incubated for 18–24 h. Finally, the diameter of the inhibition zone was measured using a black surface and transparent ruler. The results were classified as sensitive, intermediate, and resistant according to CLSI^29^. As part of the quality control system, the standard reference strain of *E. coli* ATCC 25922 was used. In addition to this, all the methods followed for microbial analysis, serology and enumeration were done following the relevant guidelines, and regulations referred to.

### Data analysis

The data obtained were entered into a Microsoft Excel spreadsheet, cleaned, coded, and then analyzed using STATA version 14, for Windows (Stata Corp. College Station, TX, USA). Cross-tabulation was used to calculate the frequencies. Logistic regression was used to assess the association between *E. coli* isolation rate and risk factors. Variables that exhibited p-values of less than 0.25 during univariable analysis and showed no evidence of collinearity were included in the multivariable analysis. The odds ratio was used to see the degree of association, and the confidence level was held at 95%. Dairy farms were considered *E. coli* positive when at least a milk sample from a cow is found positive. For the coliform count, before statistical analysis, the data were transformed to the logarithm of base ten (log10), and then one-way ANOVA was used to analyze the transformed *E. coli* count data. The significance was held at p < 0.05 in all the analyses.

### Ethical approval and consent to participate

Ambo University Research Ethics Review Committee has approved this study before it is carried out. Informed consent was obtained from each study participant before the interview and milk sample collection and participants were informed that the results of the samples would be used solely for research purposes.

## Results

### Occurrence of *E. coli* and *E. coli* O157

Out of the 556 udder milk and milk product samples, *E. coli* was isolated from 188 milk and milk product samples which is 33.8% (CI 29.9–37.9%). The occurrence of *E. coli* in bulk tank milk samples (47.4%) was significantly higher (OR 7.7, 95% CI 2.4–24.4, p 0.001) followed by udder milk samples (OR 4.5 95% CI 1.6–12.9, p 0.005) compared to yoghurt samples. When compared with yoghurt (ergo) (10.5%), though not statistically significant, the contamination with *E. coli* was marginally higher in cottage cheese 27.0% (OR 3.2, 95% CI 0.9–11.2, p = 0.066) (Table 1). In the present study, a very low prevalence (0.2%, 1/556; 95% CI 0.0–1.0%) of *E. coli* O157 was observed.

**Table 1:** Analysis of the association of *E. coli* contamination with the type samples (n = 556).

Sample types	Sample size	Number positive (%)	OR (95% CI)	p-value
Yoghurt (Ergo)	38	4 (10.5)	Ref	
Cottage cheese	40	11 (27.5)	3.2(0.9–11.2)	0.066
Cow milk	421	146 (34.7)	4.5(1.6–12.9)	0.005
Bulk tank milk	57	27 (47.4)	7.7 (2.4–24.4)	0.001
Overall	556	188 (33.8)		

### Animal-level risk factors for *E. coli* contamination

Out of the eight variables analyzed using univariable logistic regression, six of them (study area, breed of the cow, the number of lactating cows, parity, herd size, and tick infestation) satisfied the criteria to be included in the multivariable analysis after checking for co-linearity and p-value (p < 0.25). Out of the aforementioned factors, only the study area was retained in the final model. Hence, the occurrence of *E. coli* was 3.0 and 10.1 times more likely to occur in the samples collected from Ambo and Bako towns, respectively than from Holeta town (Table 2).

**Table 2:** Analysis of cow level raw milk contamination with *E. coli* and risk factors (n = 421).

Risk factors	Categories	No. positive (%)	Univariable logistic regression		Multivariable logistic regression	
			OR (95% CI)	p-value	OR (95% CI)	p-value
Study areas	Holeta	45 (20.4)	Ref		Ref	
	Ambo	65 (43.3)	3.36 (1.67, 6.75)	< 0.001*	3.36 (1.67, 6.75)	< 0.001*
	Bako	36 (72.0)	10.06 (5.00, 20.23)	< 0.001*	10.06 (5.00, 20.23)	< 0.001*
Cow age	> 10 years	76 (35.0)	Ref			
	2–5 years	69 (34.7)	2.12 (0.23, 19.37)	0.504		
	6–10 years	1 (20.0)	2.16 (0.24, 19.63)	0.495		
Breed	Local	18 (64.3)	Ref		Ref	
	Cross	6 (40.0)	2.70 (0.74, 8.43)	0.131	1.29 (0.29, 5.80)	0.740
	Exotic	122 (32.3)	3.78 (1.69, 8.43)	0.001*	0.68 (0.21, 2.22)	0.529
Parity	1st	44 (43.6)	Ref		Ref	
	3rd	11 (35.5)	1.54 (0.89, 2.68)	0.122	0.99 (0.54, 1.80)	0.967
	2nd	40 (36.0)	1.43 (0.82, 2.48)	0.204	1.13 (0.62, 2.04)	0.690
	> 4th	39 (33.3)	1.40 (0.61, 3.23)	0.426	0.99 (0.40, 2.44)	0.975
	4th	12 (20.7)	2.96 (1.40, 6.25)	0.004	2.04 (0.91, 4.60)	0.083
Stages of lactation	Middle	36 (38.3)	Ref			
	Late	23 (34.3)	1.04 (0.59, 1.83)	0.893		
	Early	87 (33.5)	1.23 (0.76, 2.01)	0.399		
No. of lactating cows	Medium	81 (45.3)	Ref		Ref	
	Small	40 (37.0)	1.40 (0.86, 2.29)	0.173	0.84 (0.36, 1.96)	0.290
	Large	25 (18.7)	3.60 (2.13, 6.09)	< 0.001*	1.40 (0.65, 3.05)	0.391
Tick infestation	No	21 (80.8)	Ref		Ref	
	Yes	152 (31.9)	9.07 (3.34, 24.6)	< 0.001*	2.79 (0.76, 10.20)	0.120
Herd size	Small	58 (40.3)	Ref		Ref	
	Medium	79 (32.1)	1.43 (0.93, 2.19)	0.104	0.96 (0.46, 2.00)	0.911
	Large	9 (29.0)	1.65 (0.71, 3.83)	0.246	1.53 (0.51, 4.58)	0.445

*Statistically significant, *OR* Odds ratio, *CI* confidence interval, *Ref* reference, *No* number.

### Farm-level risk factors for milk contamination with *E. coli*

Table 3 describes the logistic regression analysis of the farm-level risk factors associated with contamination of in milk samples with *E. coli*. Ten variables were assessed using univariable logistic regression and six passed for multivariable regression. Out of which farm hygiene, udder washing practice and the hygiene of the utensils used for milk handling were the variables retained in the final model. Consequently, the occurrence of *E. coli* was significantly higher (p < 0.05) in milk samples from farms with poor hygiene, not practicing udder washing before milking, and milk containers with poor hygienic status.

**Table 3:** Analysis of farm level raw milk contamination with *E. coli* and risk factors (n = 67).

Risk factors	Categories	No farms	No positive (%)	Univariable logistic regression		Multivariable logistic regression	
				OR (95% CI)	p-value	OR (95% CI)	p-value
Types of milk containers	Stainless steel	14	4 (28.6)	Ref			
	Plastic	53	38 (71.7)	6.3 (1.7–23.3)	0.006*	17.9 (0.8–393.1)	0.067
Farm hygiene	Good/moderate	25	2(8.0)	Ref			
	Poor	42	40 (95.2)	230 (30.3–174.2)	0.000*	132.8 (11.7–1510.4)	0.000
Management system	Intensive/semi	46	25 (54.3)	Ref			
	Extensive	21	17 (80.9)	3.6 (1.0–12.3)	0.043*	7.3 (0.3–172.4)	0.215
Herd size	Small	37	19 (51.3)	Ref			
	Large/medium	30	23 (76.7)	3.1 (1.1–9.0)	0.036*	9.06 (0.2–539.8)	0.290
Udder washing	Washing	40	18 (45.0)	Ref			
	No	27	24 (88.9)	9.8 (2.5–37.8)	0.001*	7.9(1.5–41.9)	0.015
Source of water	Pipe	59	35 (59.3)	Ref			
	River/hand dug	8	7 (87.5)	4.8(0.6–41.6)	0.154*		
Towel for udder drying	No	24	11 (45.8)	Ref			
	Collective towel	39	28 (71.8)	3.0 (1.0–8.7)	0.042*		
	Bare hand to dry	4	3 (75.0)	3.5 (0.3–39.1)	0.302		
Use of disinfectant	Yes	24	3 (12.5)	Ref			
	No	43	39 (90.7)	68.2 (13.9–34.1)	0.000*		
Hygiene of utensils	Fair to good	34	10 (29.7)	Ref			
	Poor	33	32 (97.0)	76.8 (9.2–641.5)	0.000*	37.2 (2.0–68.8)	0.015
Smoking utensils	Yes	34	10 (29.7)	Ref			
	No	33	32 (97.0)	(0.3–4.0)	0.943		

*Statistically significant, *OR* Odds ratio, *CI* confidence interval, *Ref* reference, *N*o number.

### Risk factors for the contamination of *E. coli* in bulk tank milk and milk products

The analysis for the association of bult tank milk contamination with the risk factors such as farm size, farm hygiene, farm management, and udder washing practice, use of towel, disinfection, milk containers, water source, hygiene of milking utensils, and smoking milking utensils was done. However, none of them showed significant association (p > 0.05) probably due to the small sample size (n = 57) (data not shown).

Logistic regression analysis for the association of the risk factors for the contamination of milk products with *E. coli* is presented in Table 4. Of the variables analyzed using univariable logistic regression only the source of sample was found significantly associated (p < 0.05). The other variables such as sample type, types of milk containers, environmental hygiene, and refrigerating milk products were not included in the final model.

**Table 4:** Analysis of milk products contamination with *E. coli* and risk factors (n = 78).

Risk factors	Categories	No positive (%)	Univariable logistic regression		Multivariable logistic regression	
			OR (95% CI)	p-value	OR (95% CI)	p-value
Sample type	Yoghurt	4 (10.5)	Ref		Ref	
	Cheese	11 (27.5)	3.2 (0.9–11.2)	0.066*	1.7 (0.4–7.3)	0.456
Types of milk containers	Stainless steel	1 (3.9)	Ref		Ref	
	Plastic	14 (26.9)	9.2 (1.1–74.5)	0.037*	6.5 (0.7–56.9)	0.091
Environmental hygiene	Good	2 (6.3)	Ref			
	Moderate	1 (11.1)	0.26 (0.03–2.3)	0.229		
	Poor	12 (32.4)	1.9 (0.2–23.4)	0.625		
Hand washing b/n services	Never	5 (16.7)	Ref			
	Sometimes	10 (20.8)	1.3 (0.4–4.3)	0.650		
Refrigerating milk products	Yes	4 (8.33)	Ref		Ref	
	No	11 (36.7)	6.4 (1.8–22.5)	0.004*	2.7 (0.7–11.2)	0.162
Smoking the milk utensils	Yes	7 (18.9)	Ref			
	No	8 (19.5)	1.0 (0.3–3.2)	0.947		
Source of samples	Market	5 (8.6)	Ref		Ref	
	Cafeteria	10 (50.0)	10.6 (3.0–37.7)	0.000*	10.6 (3.0–37.7)	0.000*

*Statistically significant, *OR* Odds ratio, *CI* confidence interval, *Ref* reference, *No* number.

### Milk handling and hygienic practices

Out of the 67 dairy farms involved in this study, 59.7% (40/67) farms practiced udder washing only before milking and the remaining 40.3% (27/67) did not practice udder washing at all. Also, 88.1% (59/67) and 11.9% (8/67) of the farms used pipe and river water for the sanitation of milking equipment and udder, respectively. In addition, 80.0% (4/5), 73.3% (11/15), and 57.4% (27/47) of the farms in Bako, Holeta, and Ambo towns, respectively use shared towels to dry the udder and teats of all cows. On the other hand, 35.8% (24/67) of the respondents did not practice udder drying, and 6.0% (4/67) used bare hands to dry the udder and teat (Table 5).

**Table 5:** Frequency distribution of milking-related hygienic practices in dairy farms.

Hygienic practices	Ambo	Holeta	Bako	Total
	No (%)	No (%)	No (%)	No (%)
Udder and teat washing				
Washing only before milking	28 (59.6)	9 (60.0)	3 (60.0)	40 (59.7)
No hygienic practices	19 (40.2)	6 (40.0)	2 (40.0)	27 (40.3)
Source of water for washing				
Pipe	45 (95.7)	13 (86.7)	1 (20.0)	59 (88.1)
River	2 (4.3)	2 (13.3)	4 (80.0)	8 (11.9)
Udder and teats drying				
Shared towel	27 (57.4)	11 (73.3)	4 (80.0)	42 (62.7)
Bare hand	0 (0.0)	4 (26.7)	0 (0.0)	4 (6.0)
No udder drying	23 (48.9)	0 (0.0)	1 (20.0)	24 (35.8)

### Enumeration of the coliform

The mean coliform count (CC) (log10 CFU/mL) of bulk tank milk sampled from three study areas were shown in Table 6. The overall average coliform count observed was 4.09 log10 CFU/mL, which is 4.310 ± 0.392, 4.112 ± 0.233, and 3.998 ± 0.157 for collection centers, cafeterias, and dairy farms, respectively. Among the analyzed variables, milk containers (F = 43.4, p < 0.001) and environmental hygiene (F = 35.2, p < 0.001) showed a significant association with the mean coliform count in the milk samples. Accordingly, the mean coliform count was significantly higher in those using plastic milk containers compared to stainless steel, and in poor hygienic farms compared to moderate/good environmental hygiene.

**Table 6:** Mean count coliform (log10/mL) in bulk tank milk from three the study areas.

Risk factors	Category	Range	Mean	95% CI		SE
				Lower	Upper	
Study areas	Bako	3.39–4.73	4.21	3.83	4.58	0.37
	Holeta	3.24–5.73	4.18	3.98	4.38	0.20
	Ambo	3.06–4.82	3.93	3.84	4.11	0.18
Source of sample	CC	3.23–5.73	4.31	3.91	4.70	0.39
	Café	3.38–5.23	4.11	3.89	4.35	0.23
	Farm	3.06–4.94	4.00	3.84	4.16	0.16
Milk container	Plastic	3.38–5.73	4.27*	4.15	4.39	0.12
	Stainless steel	3.06–4.63	3.48	3.31	3.65	0.17
Milker’s hand washing	Never	3.26–4.82	4.18	3.96	4.40	0.22
	Sometimes	3.06–5.73	4.03	3.87	4.20	0.17
	Mostly	3.24–4.64	4.10	3.65	4.55	0.45
Environmental hygiene	Poor	3.25–5.73	4.24*	4.11	4.371	0.13
	Good/moderate	3.06–3.90	3.678	3.48	3.916	0.25
Time range	3–4 h	3.23–5.73	4.31	3.91	4.70	0.40
	4–8 h	3.38–5.23	4.12	3.89	4.35	0.23
	Within 1 h	3.06–4.94	4.00	3.84	4.16	0.16

*Statistically significant, *CC* collection center, *CI* confidence interval, *SE* standard error.

### Antimicrobial susceptibility testing for *E. coli*

All the 42 *E. coli* isolates tested for antimicrobial susceptibility were 100% resistant to amoxicillin, ampicillin, and tetracycline. In addition, 88.1% (n = 37) of the isolates showed resistance to co-trimoxazole, 14.3% (n = 6) to amikacin, 11.9% (n = 5) to gentamycin and 2.5% (n = 1) to azithromycin. There was no observed resistance to ceftazidime, ceftriaxone, ciprofloxacin, nalidixic acid, nitrofurantoin, and norfloxacin among any of the isolated *E. coli* strains (Table 7).

**Table 7:** Overall antimicrobial susceptibility profile of *E. coli* isolates (n = 42).

Antimicrobial drugs classes	Antimicrobial drugs	Susceptible (S) No (%)	Intermediate (I) No (%)	Resistant (R) No (%)
Aminoglycosides	Amikacin (AMK)	25 (59.5)	11 (26.2)	6 (14.3)
	Gentamycin (GEN)	21 (50.0)	16 (30.1)	5 (11.9)
Beta lactams	Amoxicillin (AMX)	0 (0.0)	0 (0.0)	42 (100.0)
	Ampicillin (AMP)	0 (0.0)	0 (0.0)	42 (100.0)
Macrolides	Azithromycin (AZM)	41 (97.6)	0 (0.0)	1 (2.5)
Cephems	Ceftazidime (CAZ)	42 (100.0)	0 (0.0)	0 (0.0)
	Ceftriaxone (CTR)	42 (100.0)	0 (0.0)	0 (0.0)
Quinolones	Ciprofloxacin (CPR)	42 (100.0)	0 (0.0)	0 (0.0)
	Nalidixic acid (NAL)	42 (100.)	0 (0.0)	0 (0.0)
	Norfloxacin (NOR)	42 (100.0)	0 (0.0)	0 (0.0)
Miscellaneous group	Co-Trimoxazole (COT)	5 (11.9)	0 (0.0)	37 (88.1)
Nitrofurans	Nitrofurantoin (NIT)	42 (100.0)	0 (0.0)	0 (0.0)
Tetracycline	Tetracycline (TET)	0 (0.0)	0 (0.0)	42 (100.0)

The comparison between antimicrobial susceptibility profiles of *E. coli* isolated from different sample sources was shown in Table 8. The bulk tank milk sample isolates showed higher resistance than isolates from other sources; consequently, the multidrug resistance was 100% for *E. coli* isolates from bulk tank milk and yoghurt.

**Table 8:** Comparison of antimicrobial resistance profile of *E. coli* isolated from different sources.

Source	No. tested	Sensitive to all drugs (%)	Intermediate sensitivity (%)	Single resistance (%)	Double resistance (%)	Multiple (≥ 3) resistance (%)
Cow milk	30	0 (0.0)	0 (0.0)	0 (0.0)	3 (10)	27 (90.0)
Bulk tank milk	5	0 (0.0)	0 (0.0)	0 (0.0)	0 (0.0)	5 (100.0)
Cottage cheese	5	0 (0.0)	0 (0.0)	0 (0.0)	2 (40.0)	3 (60.0)
Yoghurt	2	0 (0.0)	0 (0.0)	0 (0.0)	0 (0.0)	2 (100.0)
Overall	42	0 (0.0)	0 (0.0)	0 (0.0)	5 (11.9)	37 (88.1)

The antimicrobial resistance pattern of *E. coli* isolates along with the antimicrobial drugs and their categorical arrangements was presented in Table 9. The multi-drug resistance (MDR) was 88.1% (37/42).

**Table 9:** Antimicrobial resistance pattern of *E. coli* isolates.

Resistivity	Antimicrobial categories	No. of isolates	%
Single drug class	AMX* AMP	4	9.52
Two drug classes	AMX* AMP TET*	1	2.4
Three drug classes	TET* AMX* COT* AMP	31	73.8
Four drug classes	AMK* AMP*AMX* COT*TET	5	11.9
Five drug classes	AMK* AMP*AMX* AZM* COT*TET	1	2.4

*AMP* Ampicillin, *AMX* Amoxicillin, *AZM* Azithromycin, *TET* Tetracycline, *COT* Co-trimoxazole, *AMK* Amikacin.

## Discussion

Most strains of *E. coli* are not pathogenic to humans, but some serotypes, such as *E. coli* O157:H7, cause severe illness. Cattle are a known reservoir for *E. coli* O157:H7 and consuming contaminated dairy products is frequently associated with outbreaks^29^.

### The overall prevalence of *E. coli* and *E. coli* O157

In the present study, E*. coli* was isolated from 33.8% (95% CI 29.9–37.9%) of the raw cow milk and milk product samples, which is consistent with the 33.9% prevalence of *E. coli* reported by Disassa et al.^24^. Higher prevalence between 44.4 and 65.0% was reported previously from Malaysia^30^, Britain^31^, and South India^32^. Lower prevalence between 20.0 and 26.0% was reported from Egypt^33^, from Tigray Ethiopia^34^, from Egypt^35^, from Bishoftu central Ethiopia^36^. The variations in prevalence of *E. coli* in milk can be attributed to dairy farming type, geographic location, milking equipment, milking technique, duration of milk transportation, and level of sanitary practices^10^. Milk contamination with *E. coli* may differ between farming systems because milking systems, milk hygiene, and handling practices are more labor and capital intensive, minimizing contact of one milker to many cows and access to clean water and other equipment^12^. The presence of *E. coli* in milk and milk products suggests poor hygienic practices during milking and milk handling, which could be a result of direct or indirect fecal contamination, posing a risk to people consuming contaminated milk.

The higher *E. coli* contamination in bulk tank milk (47.4%) compared to udder milk (34.7%), cottage cheese (27.0%) and yoghurt (10.5%) could be related to the more time and contact required for the milk to reach the bulk tank, and milk is pooled from several cows, as it was reported by Disassa et al.^24^ and Bedasa et al.^36^. Lower *E. coli* contamination in milk products such as yoghurt (ergo) and cottage cheese could be related to the fact that these products are produced from sour milk. A combination of factors such as the low pH, the production of bacteriocin, hydrogen peroxide, and ethanol by lactic acid bacteria in fermented milk has been shown to have an inhibitory effect on *E. coli* and *E. coli* O157:H7^37^. In addition, the Ethiopian cottage cheese is prepared from fermented milk after removing the butter by churning and the defatted milk is heated at a temperature between 40 and 70 °C, which could lower the bacterial load. However, the detection of *E. coli* from cottage cheese probably because of the poor hygienic handling in the households and in the open markets, which might have contributed to contamination of cottage cheese in this study. A similar scenario was also observed by Bedasa et al.^36^ in Bishoftu town who reported 40.0% and 25.7% contamination frequency of cottage cheese and yoghurt, respectively.

The comparison of *E. coli* contamination milk among the three towns showed a significantly higher prevalence in udder milk samples from Bako town (72.0%) compared to Ambo (43.3%) and Holeta (20.4%), which could be attributed to the warmer and more humid climate of the Bako town, which favors faster multiplication because *E. coli* growth increases with increasing temperature^38^. Furthermore, the more rural nature of Bako town compared to Ambo and Holeta towns may contribute to lower hygienic standards and thus higher prevalence. The results of this study indicate that cow-level risk factors, such as age, parity, breed, and stage of lactation, did not exhibit a significant association with *E. coli.* This suggests that the presence of *E. coli* is more closely linked to general hygiene conditions, rather than factors specific to cows.

*Escherichia coli* contamination was significantly higher in milk samples from farms that had inadequate hygienic standard, those using unclean milking utensils, and not properly washing udders and teats. The source of *E. coli* in the dairy farms is obviously the cow feces, and when there is lack of regular cleaning and removing the dung from the cow barn, it results in soiling the body surface of the cows including the udder and the teats. Especially when cows lay down on the dirty ground, the udder and teat come into direct contact with feces, which in the absence of proper cleaning of the udder and teat before milking could result in contamination of milk. Same way, the milk utensils used for milking and milk handling, as well as the people working on the farm, could be contaminated with the feces, which in turn could contaminate the milk. There was also a marginally higher contamination of milk in farms using plastic milk containers (45.06%) over stainless steel (26.22%). This could be because stainless steel containers are easily cleanable, which could reduce the burden of the bacteria. This finding is in accord with the reports of Disassa et al.^24^. In general, cleaning of the dairy farm environment including washing the cows and milking barn regularly, practicing good personal hygiene and washing milking and milk handling utensils are needed to avoid bacterial contamination in the current study areas.

The 0.2% prevalence of *E. coli* O157 in milk samples in the present study is lower compared the 2.9% prevalence reported by Disassa et al.^24^ from Ethiopia, and 2.8% reported from Libya^39^ and 5% from Nigeria^40^. Some scholars have reported 0.0% prevalence of *E. coli* O157 in Turkey^41^, and less than 1% in Greece^42^ and Egypt^43^. Although the occurrence of *E. coli* O157 in the present study is very low, the higher prevalence of non-O157 *E. coli,* which might bear virulence genes of other pathogenic *E. coli* forms has to be considered. It is pertinent to note that some pathogenic non-O157 serotypes of *E. coli* including O26, O103, O145, O172, O174, O113, and O111, which are none-sucrose fermenting and negative to *E. coli* O157 latex kits exists^44^*.* Some of these non-O157 groups have been associated with infections in humans^45,46^. Karns et al.^47^ suggested that the presence of *E. coli* in raw milk also represents a significant public health risk.

### Handling practices of milk along the dairy milk supply chains

Hygienic practices are major pathways to producing safe and quality products for consumers thereby reducing microbial contamination^48^. Therefore, the source and type of water used for washing the hand and milking utensils have a profound effect on microbial contamination of the milk. In the present study, most farmers (88.1%) use pipe water for cleaning milk utensils and udder compared to the 56.6% report from Wolayta Sodo, southern Ethiopia^49^. However, 40.3% of the producers did follow hygienic practices such as udder and hand washing before and after milking, which is much higher than the 3.7% report in Jimma, southwestern Ethiopia^50^. The majority of those who practice washing the udder (59.7%) wash only before milking and none of them had an individual towel for each cow for wiping the udder and teats. However, Kuma et al.^51^ have reported the use of individual towels by 3.8% and 3.4% of the producers in Holeta and Sebeta towns of Central Ethiopia, respectively. A variety of factors, such as poor handling and storage conditions, contaminated water, and a lack of adequate temperature control, can all contribute to unsafe food. In extreme cases, such safety issues can jeopardize a country's food security^52^. As a result, producing good hygienic quality milk for consumers necessitates good hygienic practices such as washing milkers' hands, cleaning the udder, and using individual towels during milking and handling before delivery to consumers or processors^53^.

### Enumerations of coliform

The coliform count is one of the most used methods to estimate the microbial load in milk and milk products. The increment of bacterial load could be attributed to the contamination of the milk from production to consumer by different factors like the pooling of milk from different sources together with unhygienic handling. The overall mean coliform count detected in the bulk tank milk samples in the current study was 4.09 log10 CFU/mL, which is relatively lower than the 6.54 ± 1.53 log10 CFU/m count reported by Aliyo and Teklemariam^54^ from the southern pastoral areas of Ethiopia. Coliform count in milk managed under poor hygienic conditions was significantly (p < 0.05) higher than the milk under good and moderate hygienic conditions suggesting that the practice of production of milk under poor hygienic conditions favors the contamination with coliform and could be responsible for the high observed counts. The overall value of coliform counts observed in the current study was much higher when compared with the recommended values given by the American Public Health Service: < 100 CFU/mL for Grade A milk and 101–200 CFU/mL for Grade B milk^55^.

Generally, the presence of high numbers of coliform in milk indicates that the milk has been contaminated directly or indirectly with fecal materials and it is an index of the hygienic standard used in the production of milk. Unclean udder and teats due to unhygienic practices and cows with subclinical coliform mastitis can also lead to an elevated coliform count in raw milk^55^. These high coliform counts show that the milk produced by dairy producers and milk collection centers and distributed to cafeterias or consumers in the study areas is of poor bacteriological quality and thus the possibility of contracting infections or intoxication from milk-borne pathogens on the consumption of raw milk is probably high.

### Antimicrobial susceptibility profile of *E. coli* and *E. coli* O157

This study revealed that most of the isolates showed MDR to the antimicrobials most frequently used such as amoxicillin, ampicillin, and tetracycline. This could be due to the prolonged and frequent usage of these drugs in the health sector or the cross-resistance within the beta-lactamase group. Thus, all *E. coli* and *E. coli* O157 isolate in the present study exhibited resistance to at least two of the thirteen antimicrobial drugs used. Bedasa et al.^36^ from Ethiopia reported 100% MDR and Dulo et al.^56^ also reported a 100% resistance of isolates to 2 or more drugs. Similar MDR patterns were also reported by Atnafie et al.^13^, from other sample sources. The rapid development of MDR may severely hamper the effectiveness of treatments and the ability to control infectious diseases in both animals and humans^57^. Therefore, the development of antimicrobial resistance among bacteria like *E. coli* poses an important public and animal health concern since they are commonly used in the treatment of patients in public health and veterinary practices in Ethiopia.

The detection of *E. coli* O157 in milk and dairy products as well as its antimicrobial resistance have been reported in several studies from around the world as it is a growing concern^58–63^. Depending on the level of hygiene standards and milking and handling procedures, and possibly the detection method followed, the incidence may differ between nations and among dairy farms. *Escherichia coli* O157, however, is a potential threat to food safety and a public health hazard because it can cause serious illnesses like hemorrhagic colitis, and hemolytic uremic syndrome, which can be fatal^64^. As a result, the reports implicate the importance of maintaining adequate farm hygiene and effective hygienic methods throughout milking, handling, and processing to avoid contamination of milk and milk products. Proper cleaning of milking equipment and utensils, ensuring the hygiene of milk handlers, utilizing high-quality water for cleaning, and maintaining udder health are all critical hygiene practices.

As a limitation, surveillance, and antimicrobial susceptibility of *E. coli* isolates among food handlers using stool samples for comparisons with milk, milk product isolates, and enhanced public health intervention was not done. The small number of farms and bulk tank samples made it difficult to assess the risk factors for contamination of milk using multivariable regression. Due to the absence of flagella (H) antigen latex kit, the *E. coli* was tested only for somatic (O) antigen. In addition, molecular and other advanced studies were not carried out due to the lack of materials and suitable laboratories.

## Conclusions

In this study, the prevalence of *E. coli* was higher, while that of *E. coli* O157 was too low. The detection of *E. coli* in milk and milk products implies the presence of fecal contamination which suggests a potential risk to public health and food safety. In the study areas, inadequate sanitary measures in dairy farms, uncleaned milking utensils and containers, and unhygienic milking and milk handling activities were the identified risk factors. The coliform count was higher than the international acceptable limit which indicates the poor quality of raw milk that results in short shelf life. Importantly, the occurrence of MDR *E. coli* shows a risk to public health, as well as animal health and production. Thus, improving hygienic practices such as washing hands, udder, and teat before and after milking, avoiding the use of shared towels for drying different cows’ udder and teats, and use of clean containers for storage and transportation of milk and milk products are recommended. Moreover, public need to be aware of hygienic milk handling and avoidance of raw milk consumption. The indiscriminate use of antimicrobial drugs should be avoided in both humans and animals to safeguard the public from the antimicrobial-resistant pathogens.

## Data Availability

The datasets supporting the conclusion of this article are included within the article.

## Abbreviations

AMR: Antimicrobial resistance
ATCC: American Type Culture Collection
CFU: Colony forming units
CI: Confidence interval
EMB: Eosin-Methylene-Blue agar
MDR: Multidrug resistance
